# ABCDs of the Relative Contributions of Pseudomonas aeruginosa Quorum Sensing Systems to Virulence in Diverse Nonvertebrate Hosts

**DOI:** 10.1128/mbio.00417-22

**Published:** 2022-03-21

**Authors:** Alejandro Vasquez-Rifo, Jamie Cook, Deborah L. McEwan, Dania Shikara, Frederick M. Ausubel, Francesca Di Cara, Zhenyu Cheng

**Affiliations:** a Program in Molecular Medicine, University of Massachusetts Medical School, Worcester, Massachusetts, USA; b Department of Microbiology and Immunology, Dalhousie University, Halifax, Nova Scotia, Canada; c Department of Molecular Biology, Massachusetts General Hospital, Boston, Massachusetts, USA; d Department of Genetics, Harvard Medical School, Boston, Massachusetts, USA; School of Medicine, Oregon Health & Science University

**Keywords:** *Pseudomonas aeruginosa*, quorum sensing, broad host range, opportunistic pathogen

## Abstract

Pseudomonas aeruginosa is an opportunistic bacterial pathogen that exhibits pathogenicity in an unusually broad range of plants and animals, and it is of interest to study the roles of particular virulence-related factors in diverse hosts. The production of many P. aeruginosa virulence factors is under the control of a quorum sensing (QS) signaling network, which has three interconnected branches that engage in intricate cross talk: Las, Rhl, and MvfR. Because there has been no systematic comparison of the roles of the three QS systems in mediating P. aeruginosa virulence in various hosts, we compared the virulence of wild-type (WT) P. aeruginosa PA14 and a set of isogenic PA14 QS in-frame deletion mutants in four selected hosts, the reference plant Arabidopsis thaliana (Arabidopsis), the crop plant Brassica napus (canola), the nematode Caenorhabditis elegans, and the fruit fly Drosophila melanogaster. The first letters of the selected host genera, A, B, C, and D, inspired the title of this article and indicate that this work lays the groundwork for future elucidation of the specific roles of each QS branch in mediating virulence in diverse hosts.

## OBSERVATION

The large genome of Pseudomonas aeruginosa encodes a network of regulatory systems that contribute to its metabolic versatility, adaptability to environments, and broad host range as a pathogen (1–3). The P. aeruginosa quorum sensing (QS) signaling network consists of three branches, each of which plays an important role in regulating virulence (4–8). To help elucidate the specific contribution of each QS branch to pathogenesis, we assembled a comprehensive set of isogenic in-frame deletion mutants in QS genes in P. aeruginosa PA14, including the single Δ*lasR*, Δ*lasI*, Δ*rhlR*, Δ*rhlI*, and Δ*mvfR* mutants and different combinations of higher order Δ*lasR* Δ*rhlR*, Δ*lasR* Δ*mvfR*, Δ*rhlR* Δ*mvfR*, and Δ*lasR* Δ*rhlR* Δ*mvfR* mutants. We used this set of mutants to compare their virulence to wild-type (WT) PA14 in four P. aeruginosa hosts. Importantly, none of the mutant strains grew at a statistically significant slower rate than wild-type PA14 as determined by growing four replicate cultures of each strain in LB medium for approximately 20 h at 25°C in an automated plate reader.

**FIG 1:**
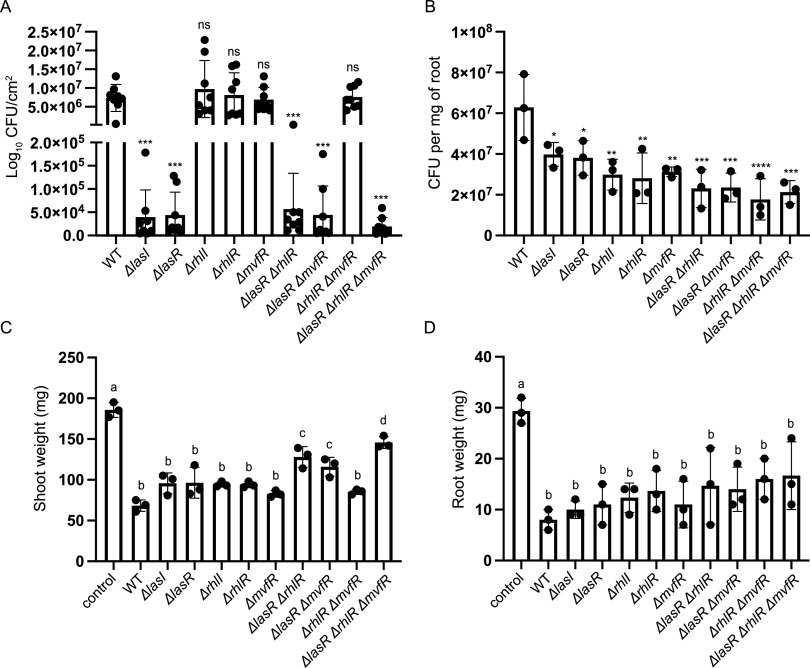
Plant and bacterial growth during P. aeruginosa infections. (A) *In planta* growth of P. aeruginosa PA14 WT and QS mutants 3 days after infiltration in 5-week-old *Arabidopsis* (ecotype Col-0) leaves measured by log_10_ CFU/cm^2^ leaf area. All *las* mutants showed significantly decreased growth compared to WT (*P* < 0.001). (B) Colonization of canola roots by WT and QS mutants measured on day 5 postinfection. All QS mutants showed modest but highly significant reduction in root association compared to that of the WT. Canola root (C) and shoot (D) weights recorded 5 days postinfection with WT or QS mutants. In panels C and D, all strains (WT and QS mutants) showed significant shoot and root weight loss compared to that of the uninfected control. Three higher order QS mutants (Δ*lasR* Δ*rhlR*, Δ*lasR* Δ*mvfR*, and Δ*lasR* Δ*rhlR* Δ*mvfR*) had modest but significant alleviation of shoot weight loss. Error bars represent standard deviation of 10 (A) or 3 (B to D) independent biological replicates. Independent sample means were analyzed using a one-way ANOVA with a Dunnett’s multiple comparison *post hoc* test to determine differences between samples. For *in planta* (A) and root-associated (B) CFU counting, significance is indicated as follows: *, *P* < 0.05; **, *P* < 0.01; and ***, *P* < 0.001. For plant tissue weight data (C and D), significant differences (*P* < 0.05) are reported as different letters.

Crop plants can be naturally infected by P. aeruginosa (9), and ornamental plants can serve as a reservoir for P. aeruginosa infection of hospitalized immunocompromised patients (10). We used a leaf infiltration assay in *Arabidopsis* (11) to compare the virulence of the WT and QS mutants. Plants infiltrated with a P. aeruginosa strain that has a functional Las QS system (WT and Δ*rhlR*, Δ*rhlI*, Δ*mvfR*, or Δ*rhlR* Δ*mvfR* mutants) showed high bacterial growth in leaves, whereas mutants lacking a functional Las QS system (Δ*lasR*, Δ*lasI*, Δ*lasR* Δ*rhlR*, Δ*lasR* Δ*mvfR*, and Δ*lasR* Δ*rhlR* Δ*mvfR* mutants) exhibited dramatically decreased growth (Fig. 1A).

We also tested the virulence of the QS mutants in a *Brassica napus* root colonization model (12). Roots are in close contact with pathogens in the soil, and bacteria that colonize plant roots need to partially nullify root defense responses (13). In contrast to the *Arabidopsis* leaf infiltration model in which Las-mediated QS is a key virulence determinant, mutation of any of the QS branches resulted in a consistently modest but highly statistically significant reduction in root colonization, with the double and triple mutants exhibiting more pronounced colonization defects with higher statistical significance (Fig. 1B). Monitoring virulence by assessing the growth of the host showed that the loss of canola shoot weight caused by colonization of PA14 WT was partially abrogated in the Δ*lasR* Δ*rhlR*, Δ*lasR* Δ*mvfR*, and Δ*lasR* Δ*rhlR* Δ*mvfR* mutants (Fig. 1C). All of the mutants exhibited consistently modest increases in root weight compared to PA14 WT that were not statistically significant (Fig. 1D). A previous publication reported that a Δ*rhlI* mutant and a Δ*rhlI* Δ*lasI* mutant, but not a Δ*lasI* mutant, exhibited partially and completely disrupted root-associated growth in *Arabidopsis* and sweet basil, respectively (14), suggesting that different QS systems may be involved in root colonization in different hosts.

**FIG 2:**
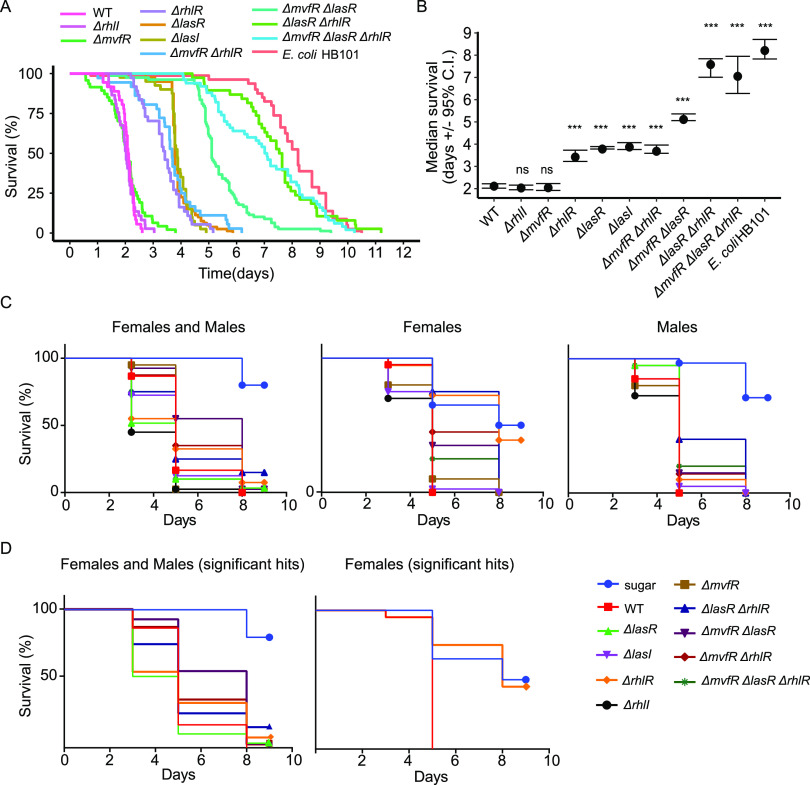
Survival of C. elegans and D. melanogaster exposed to P. aeruginosa PA14 WT and QS mutants. (A) Survival curve of adult worms (in days) following infection with the indicated P. aeruginosa PA14 WT and QS mutants. E. coli HB101 was included as a negative control. Pairwise comparisons of the survival curves between each strain and the WT were done using the log rank test. The test *P* values were all significant (*P* < 0.0001) except for Δ*mvfR* and Δ*rhlI.* (B) Median survival estimate (in days with confidence interval [CI]) for the survival curves shown in panel A. (C) Survival curves of mixed, female, and male D. melanogaster following infection with the P. aeruginosa PA14 WT and QS mutants. Uninfected sugar-fed flies were included as controls. (D) Survival differences were analyzed by Kaplan-Meier and log rank analysis. Only survival curves of mixed and female flies for WT and for statistically different (*P* < 0.05) QS mutants are shown. No QS mutants were statistically different from WT in infected male flies.

The nematode Caenorhabditis elegans has been used extensively as a model host to study bacterial virulence (15). Here, we monitored P. aeruginosa-mediated killing of C. elegans using a semi-automated life span device (16) (Fig. 2A and B). We found that the Δ*mvfR* or Δ*rhlI* mutations did not impair the ability of PA14 to kill nematodes, whereas all of the other mutants exhibited reduced virulence, resulting in longer nematode lifespans (Fig. 2A). We observed that both the Las and Rhl QS branches are required for full virulence. In contrast, the MvfR QS system appears to play a minor role in PA14-mediated killing. Only in one mutant (Δ*lasR* Δ*mvfR* mutant) did the loss of *mvfR* result in reduced virulence. Similar to other reports (17, 18), we observed that the Δ*rhlI* and Δ*rhlR* mutants exhibited very different phenotypes with respect to their ability to kill C. elegans. More specifically, the Δ*rhlI* mutant was indistinguishable from WT PA14, whereas the Δ*rhlR* mutant exhibited significantly impaired killing ability. This latter observation is consistent with a report showing that in addition to C4-HSL synthesized by RhlI, RhlR can also be activated by a ligand that relies on PqsE (19).

The fruit fly Drosophila melanogaster has also been used to study host-bacterial interactions (18, 20, 21). We tested *W^1118^* adult flies for their ability to survive infection caused by feeding flies the PA14 QS mutants. The resulting survival curves are shown in Fig. 2C. To aid data visualization, the sugar control, WT infection, and QS mutants that were significantly different from WT are plotted in Fig. 2D. When female or male flies were raised and infected separately, all of the flies died 5 days postinfection upon infection with WT PA14 or the Δ*rhlI* mutant, whereas all the other QS mutants showed a reduction in virulence (Fig. 2D). In particular, the female flies infected with the Δ*rhlR* mutant showed survival rates similar to those of the uninfected control, whereas other mutants containing the Δ*rhlR* mutant demonstrated reduced virulence that did not reach statistical significance (Fig. 2D). Conversely, when males and females were raised and infected in the same vial, they showed a slight but significant increase in survival to infections with the Δ*lasR* Δ*rhlR*, Δ*lasR*, and Δ*mvfR* mutants. The higher order mutants did not demonstrate further abrogated virulence than the Δ*rhlR* mutant in our study, which is consistent with a previous observation that in a similar feeding model, RhlR but not RhlI or LasR contributed to P. aeruginosa evasion of *Drosophila* immunity (19, 21). Interestingly, sexual dimorphism in infection has been reported previously and was shown to be dependent on the specific environment, as well as host and pathogen genetic backgrounds (22).

To our knowledge, this is the first study to systematically investigate the virulence of P. aeruginosa QS mutants representing all three branches of the P. aeruginosa QS network in multiple plant and invertebrate hosts. Interestingly, no consistent pattern of the role of the different QS systems in virulence emerged from these studies, suggesting that the “state of virulence” is both host and/or infection model dependent. We would argue that these results are consistent with previous results (15), which show that P. aeruginosa is a broad range multihost pathogen that has a variety of virulence mechanisms that allow it to physiologically adapt to particular host environments.

### Mutant compiling and construction.

The Δ*lasR* (DH164), Δ*rhlR* (DH2742), Δ*mvfR* (DH1110), Δ*lasR* Δ*rhlR* (DH2944), and Δ*lasR* Δ*mvfR* (DH1111) mutants in the P. aeruginosa strain PA14 (DH123) background (the labels in the brackets represent the lab stock numbers of these strains) were constructed previously and kindly provided by D. Hogan (23). The Δ*lasI* and Δ*rhlI* mutants of PA14 have been described (18, 21), and the Δ*rhlR* Δ*mvfR* and Δ*lasR* Δ*rhlR* Δ*mvfR* mutants were constructed using previously described protocols (18, 21). All mutations were confirmed by diagnostic PCR.

### Host infection assays.

All infection assays using the four selected hosts were performed using previously published protocols. *Arabidopsis* (ecotype Col-0) leaf infiltration and canola root colonization experiments were carried out as described previously (13, 14). For the C. elegans infection, the life span and slow killing assay were performed using a C. elegans Lifespan Machine as previously described (16). An oral infection model of *Drosophila* (24) was used to measure fly survival. In brief, 20 flies (age-matched males and females together or separate) were cultured on filter paper soaked in 5% sucrose or 5% sucrose containing P. aeruginosa WT or individual mutant strain (all optical density at 600 nm [OD_600_] = 3). Flies were transferred to fresh vials every 2 days, and the number of dead flies was determined daily.

### Statistical analysis.

For *in planta* and root-associated CFU counting and plant tissue weight data, independent sample means were analyzed using a one-way analysis of variance (ANOVA) with a Dunnett’s multiple comparison *post hoc* test to determine differences between samples. Significance was measured at *P* < 0.05, and significant differences were reported as different letters. The curated C. elegans survival data obtained from the Lifespan Machine were analyzed using R 3.5 (survival and survminer packages), and the log rank test was used to evaluate differences in worm survival. *Drosophila* survival data were analyzed in Prism 9, and statistical significance between the different survival curves was determined by Kaplan-Meier and log rank analysis.
